# A Low-Cost Paper-Based Device for the Colorimetric Quantification of Bilirubin in Serum Using Smartphone Technology

**DOI:** 10.3389/fchem.2022.869086

**Published:** 2022-07-07

**Authors:** Brittany AuYoung, Akshay Gutha Ravichandran, Divykumar Patel, Nisarg Dave, Achal Shah, Brianna Wronko-Stevens, Franklin Bettencourt, Reshma Rajan, Nidhi Menon

**Affiliations:** ^1^ Division of Product Development, Group K Diagnostics, Philadelphia, PA, United States; ^2^ Manufacturing Department, Group K Diagnostics, Philadelphia, PA, United States; ^3^ Group K Diagnostics, Philadelphia, PA, United States

**Keywords:** diagnostics, paper microfluidics, object detection, image processing, bilirubin

## Abstract

Total bilirubin values have been used as a potential marker to pre-screen and diagnose various liver-based diseases such as jaundice, bile obstruction, liver cancer, etc. A device known as KromaHealth Kit, composed of paper and an acrylic backbone, is developed to quantify total bilirubin in human serum using image processing and machine learning technology. The biochemical assays are deposited on absorbent paper pads that act as reaction zones when serum is added. A dedicated smartphone app captures images of the colorimetric changes on the pad and converts them into quantitative values of bilirubin. The range of bilirubin concentration that can be quantified using the device ranges from 0.5 mg/dl to 7.0 mg/dl. The precision, limit of detection, interference analysis, linearity, stability, and comparison with a predicate are studied in this paper in accordance with clinical and laboratory standards institute. The results indicate that the KromaHealth Kit can be used as an inexpensive alternative to conventional bilirubin testing in clinical settings. With its level of precision, ease-of-use, long shelf-life, and short turnaround time, it will prove to be invaluable in limited-resource settings.

## 1 Introduction

Bilirubin has continued to prove its importance in both clinical research as well as in the field of diagnostic laboratory testing. Normal bilirubin concentrations range from 0.30 to 1.20 mg/dl. Elevated levels of bilirubin can indicate jaundice (icterus), hepatitis, cirrhosis, Gilbert’s syndrome, Crigler-Najjar syndrome, and other serious underlying conditions (Clarke, 2011). When bilirubin is monitored in close correlation with other liver enzyme tests, it can be useful in assessing whether the elevated bilirubin can be attributed to hemolysis or other non-hemolytic liver disorders (Gourley, 1997; Kamath, 1996). It is an important biomarker, with value as a diagnostic and prognostic factor for a plethora of disease pathologies.

Conventional methods of bilirubin detection rely on spectrophotometry, chemiluminescence, chromatography, and capillary electrophoresis (Ngashangva et al., 2019; Rawal et al., 2020). These commonly used techniques are time-consuming, require expensive and complex equipment, and skilled labor. As an alternative, various types of biosensors have been developed for bilirubin, ranging from electrochemical to optical sensors (Ngashangva et al., 2019; Rawal et al., 2020; Shoham, 1995)^,^ (Ellairaja et al., 2017; Hooda et al., 2017). However, despite low costs of manufacturing and rapid detection, development and material costs for these biosensors are high, with poor reproducibility and a short shelf-life. The majority of these biosensors use enzymatic reactions, which can contribute to the aforementioned limitations (Mano and Edembe, 2013). Alternatively, miniaturization of the conventional diazo-based chemical principle on paper-based colorimetric platforms have yielded promising results (Tan et al., 2020a; Tan et al., 2020b). Paper microfluidic sensors are inexpensive, fast, and easy-to-use in point of care (POC) and clinical settings. Previous studies have shown successful quantification using paper-based colorimetric and fluorescent diagnostics and optical detection (Chu et al., 2020; Mutlu et al., 2017; Hong and Chang, 2014; Alawsi et al., 2021). However, earlier colorimetric paper-based devices for bilirubin detection are mostly capable of providing semi-quantitative results and many are only capable of detecting hyperbilirubimia (Tan et al., 2020a; Tan et al., 2020b; Keahey et al., 2017). KromaHealth Kit provides accurate quantification of clinically relevant bilirubin levels in serum. This is useful for early diagnosis of elevated bilirubin and for continuous monitoring of bilirubin levels when following treatment plans.

KromaHealth Kit was developed to address the drawback that comes with colorimetric chemical tests. For the detection of bilirubin, the kit utilizes the Diazo-dye method stabilized on a paper-based device (Rand and Pasqua, 1962; Fossati et al., 1989; Rutkowski and deBaare, 1966). Total bilirubin found in the serum reacts with the diazo reagent in the presence of accelerators to form azobilirubin under acidic conditions. The intensity of the color is quantified using an iOS application in conjunction with an iPhone camera, and an engineered box to standardize lighting. The device has potential for use in clinical settings in low-income, limited-resource areas, specifically for long-term monitoring of patients being treated for elevated bilirubin. This study focuses on analyzing the precision, effect of interferents, limits of detection, linearity, stability, and equivalence to a predicate.

## 2 Methods

### 2.1 Device and Box Manufacturing

The device consists of an acrylic backbone manufactured using a laser cutting machine (BOSS LS1630). Alpha cotton linter cellulose is the material used for the absorbent paper-pads (I.W. Tremont) and they are securely embedded in the open acrylic panels, acting as the reaction zones. Accompanying the device is an engineered closed acrylic box, also manufactured using the laser cutting machine. The box acts as a barrier to keep out ambient light and minimizes external interference. The box contains an inbuilt light source and a dedicated indent to keep the device optimally after the sample is added. The lid contains a holder to place the phone with the camera for image processing.

### 2.2 Preparation of the Reagents

The chemical principle behind the assay is a modified version of the Jendrassik-Grof Diazo method (Garber, 1981; Jendrassik and Gróf, 1938). A solution of 10.5 mg/ml sulfanilic acid (Sigma #822338) in 1.5% v/v 1M HCl(aq), 4.16 mg/ml sodium nitrite (Alfa Aesar #A18668) in DI water and accelerants 230 mg of caffeine (Sigma #27602), 1.94 g sodium acetate (Sigma #S2889), and 695.6 mg sodium benzoate (Sigma #109169) in 15 ml DI water, are used to prepare the reagent solution. The sulfanilic acid, sodium nitrite and accelerants are mixed in 4 to 1 to 4 ratios respectively and 5 μl of this reagent is deposited and dried on the paper pads on the device.

### 2.3 Procedure for the Assay, iOS App Development and Image Processing

The patient’s serum (15 μl) is pipetted onto the paper pads and allowed to react with the reagents for 50 min. The device is then placed in the indent inside the box. The phone is placed on top of the box so that the camera is aligned with the viewing port to take a picture of the device after 50 min using the KromaHealthKit app. The app takes a picture of the device, processes the image, and returns a predicted concentration of Bilirubin based on the analysis of the image.

The KromaHealthKit app was developed to capture images, transfer the images to the cloud for processing, and then display results back to users. The app was developed on the iOS 14.3 platform using Swift, which is Apple’s open-source mobile app development framework (Swift. Apple Inc, 2015) and should therefore function adequately on iPhone 7 and higher devices. The app’s object detection and analysis back-end is hosted on Amazon Web Services (AWS) and the Application Program Interface (API) to push and pull data from AWS was developed using Node.js (Dahl, 2009). Python was used to perform the object detection and image analysis for the application (Garber, 1981). MongoDB was chosen as the database to store app data. Supplementary Figure S1 shows the stack overview.

After the app captures the image of the device, it is uploaded to the AWS S3 data storage bucket for the app. After uploading the image, the backend API executes the python routine on the image. A methodology was developed using computer vision (specifically OpenCV toolkit) to detect the region of interest (ROI) in the given images (Bradshi, 2000). The ROI in our case is the subsection of the paper pads on the assay devices where the serum was placed and therefore where the assay chemistry will have taken place. The ROI sub-image is duplicated into the RGB, XYZ, LAB, and HSV color spaces, and statistical features are calculated on all those color spaces, see Supplementary Figures S2, S3 for the workflow. A predictive model was trained using samples of known concentrations and the previously mentioned statistical features collected for the ROI sub-images using the Python library Sci-kit Learn (Pedregosa et al., 2011). Using this model, the app can predict a patient’s bilirubin levels from the assay device images.

For the following studies, only a single panel (the right panel) of each device was used for testing to optimize image processing and as proof-of-concept, and the iPhone 7 was used as the baseline hardware.

### 2.4 Precision Studies

A reproducibility study was performed to determine multi-site precision of the device. The protocol for the study was developed using the guidelines from CLSI document EP05-A3 (McEnroe, 2014). Multiple lots of devices in multiple sites with different operators were used to perform this study. Serum samples with spiked bilirubin were used for this test. The same concentrations of bilirubin were used for the entire 5-day study at each site. Three different laboratory sites were used to conduct this study. The five bilirubin concentrations used were 0.4, 0.2, 0.98, 1.5, and 2.0 mg/dl, samples P1 through P5, respectively. The same samples were used in all three sites to carry out a 5-day study with five sample replicates each day. Devices from a single lot are used to perform the study at one site.

A repeatability study was performed to determine within-laboratory precision of the device. The protocol for the study was developed using the guidelines from CLSI document EP05-A3. Multiple lot devices in a single site were used to perform this study. Serum samples (Lee BioSolutions) with spiked bilirubin were used in this study. In total, 10 different samples were used to finish this study, where two samples were tested each day for over the span of 20 days. The concentrations that were used for testing are 0.2 mg/dl (Sample A); 0.4 mg/dl (Sample B); 0.97 mg/dl (Sample C); 1.5 mg/dl (Sample D); 2.0 mg/dl (Sample E); 3.3 mg/dl (Sample F); 4.1 mg/dl (Sample G); 5.6 mg/dl (Sample H); 6.5 mg/dl (Sample I) and 7.4 mg/dl (Sample J). The same sample was used for a 20-day experiment with two runs each day and two replicates. The two daily runs were performed in the morning and evening (or at least 4 h apart). The same lot and same conditions were used while testing a given sample.

### 2.5 Interference Studies

The interference test is designed to determine whether the constituents of serum could potentially interfere with the test results of the device. The concentrations of interferents and analytes used for this protocol are prepared as per the guideline given in CLSI-EPO7 (interference testing with clinical chemistry) and CLSI-EP37 (supplemental tables for interference testing in clinical chemistry) (McEnroe et al., 2018). The total number of iterations for each interference study is five trials. The previously reported (in literatures) potential interferent for colorimetric detection of bilirubin in serum are hemoglobin, triglycerides, ascorbic acid, acetaminophen, acetylsalicylic acid, intralipid and ibuprofen. The recommended test concentrations of all the interferents are as follows: hemoglobin 1,000 mg/dl; triglyceride 1,500 mg/dl; ascorbic acid 1.75 mg/dl; acetaminophen 5.20 mg/dl; acetylsalicylic acid 3.00 mg/dl; ibuprofen 21.9 mg/dl.

The bilirubin solution is prepared by spiking the serum. All the clinical serum samples used to perform this study are purchased from Lee Biosolutions. The vendor also provides a sheet which contains the values (concentration) of bilirubin and the value of interferents present in the pooled serum. To test for interference, the concentration of the analyte in the serum is tested at two different concentrations, where one is considered as the “Minimum” test concentration and the other as “Maximum” test concentration. These concentrations were determined by considering the upper and lower limits of the reference interval (Values taken from EP37). The minimum and maximum bilirubin test concentrations were 0.4 and 2.0 mg/dl, respectively. The interferents tested are Hemoglobin, Triglyceride, Ascorbic Acid, Acetaminophen, Acetyl salicylic acid and Ibuprofen. The interference study was divided into two sections: a low concentration study and a high concentration study. The low-test concentration use is 0.4 mg/dl, and the high concentration is 2.0 mg/dl.

### 2.6 Limits of Detection

Limit of Blank (LoB) and Limit of detection (LoD) protocol were prepared with reference to CLSI-EP17 A2 (Pierson-Perry, 2012). The blank sample has an analyte concentration lower than the detection limit and was prepared from the serum sample by diluting it further. The LoB is termed as the highest concentration that could be observed with a blank sample. Two reagent lots were used to do the LoB testing and the test was carried out over 3 days. Four blank samples were prepared to perform the experiment. The concentration of each of these samples was 0.08 mg/dl (Blank 1), 0.11 mg/dl (Blank 2), 0.14 mg/dl (Blank 3), and 0.17 mg/dl (Blank 4). Each sample was tested for two replicates in each reagent lot.

For the lower LoD test, f ive samples were prepared with the suspected lowest level of detection possible. Each sample was tested for five replicates (five devices) for a span of 5 days. LoD is defined as the lowest concentration of the analyte that can be detected consistently. Two reagent lots were used for testing. Three replicates were tested for Lot A, and two for Lot B. The concentrations of these samples were 0.27 mg/dl (Sample 1), 0.3 mg/dl (Sample 2), 0.33 mg/dl (Sample 3), 0.35 mg/dl (Sample 4), 0.37 mg/dl (Sample 5).

### 2.7 Linearity

The linearity test guideline describes the statistical process for determining the linearity of a quantitative measurement procedure. The primary objective is to determine the concentrations at which a method becomes nonlinear and the extent of nonlinearity. The linearity test protocol and guideline were prepared according to CLSI EPO6-A (McEnroe, 2020). Serum samples of various concentrations of bilirubin were used for this study. Seven samples were selected in which the concentrations of each sample were kept equidistant or such that there is an observable relationship between the samples. The concentrations tested were 0.2, 1.0, 1.8, 2.6, 3.4, 4.2, 5.0 mg/dl which is represented as sample 1, 2, 3, 4, 5, 6, and 7, respectively. The samples were tested in the KromaHealth device. Each sample was tested in duplicates. The known concentration and its corresponding results were noted for further analysis.

### 2.8 Predicate Device Comparison and Bias Estimation

This study is conducted to compare the performance of KromaHealth Kit with a comparative measurement procedure (predicate device). The test protocol and guidelines were prepared using CLSI EP09 (Budd, 2018). In this section Kromahealth kit results were compared to Roche cobalt c311 results, termed as the predicate method. All the clinical samples were collected from Access Biologics, CA with known concentrations of bilirubin (predetermined using the predicate device). All 57 samples were tested to perform procedure comparison. The study was done over the span of a week. Multiple test lots were used to perform this protocol.

### 2.9 Shelf-Life Study

The stability of the device is evaluated as per the CLSI guidelines in EP25-A (Pierson-Perry, 2009). The devices, once manufactured and coated with reagents, are individually packed and sealed in metalized mylar pouches with a silica gel and humidity strip inside. A 6-month study of the shelf-life of the devices with the assay was tested using spiked serum samples, once every 4 weeks. The concentration of the serum samples was 1.5, 4.0 and 7.0 mg/dl. Duplicates were tested for each concentration with each of the two lots of devices. Spiked serum samples of the concentrations listed above were prepared and stored as aliquots for each test in −20°C and thawed at room temperature before each test.

## 3 Results and Discussion

### 3.1 KromaHealth Kit Device and Box Engineering

**FIGURE 1:**
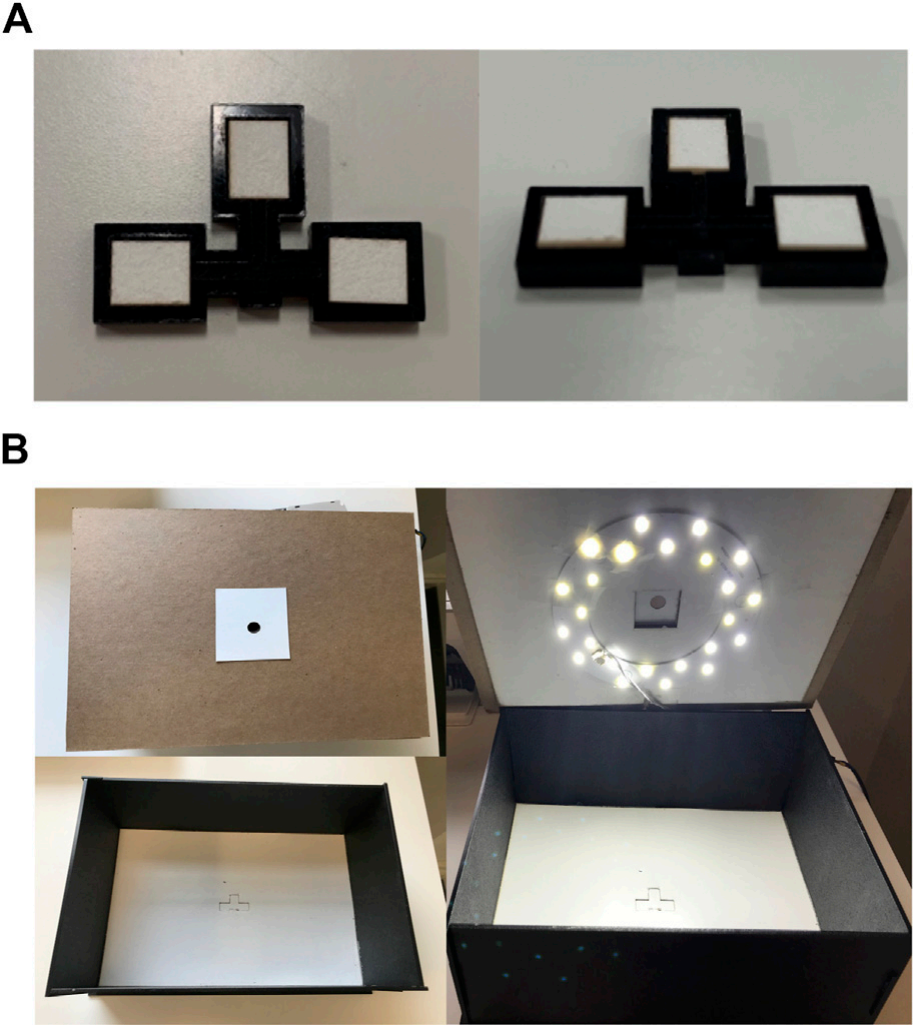
Device and box design parameters. **(A)** The top and side views of the device, with three reaction panels consisting of the alpha cotton linter cellulose absorbent pads. **(B)** The top cover of the box has a pinhole for the phone camera, the inside of the box consists of an indent for the device to be placed for capturing the image, and the lighting within the box standardizes the lighting for the image process analysis.

The device consists of an acrylic backbone, with three panels where absorbent paper pads can be tightly fitted, as shown in Figure 1A. The device consists of a barcode arm which is 12 mm × 8 mm, each panel of the device is 10.5 mm × 9 mm. The device is 3 mm thick. The paper piece is 7.5 mm × 6 mm and 0.7 mm thick. The box manufactured to quantify bilirubin concentration was developed after optimizing the width, height, light placement, and coverage to produce the best quantification of colorimetric data from the images. The box is 270 mm × 205 mm on the base and the lid is 275 mm × 210 mm. The box is 112 mm in height. The box is pictured in Figure 1B and shows the different features that enable standardized image processing for each device. The top view of the outside and inside of the box can be seen, as well as the lid with the lighting. The cost to manufacture a single device, including reagents and labor is $1.44 and for the box is $50, including the LED light ring and full assembly.

### 3.2 Colorimetric Quantification of Bilirubin

**FIGURE 2:**
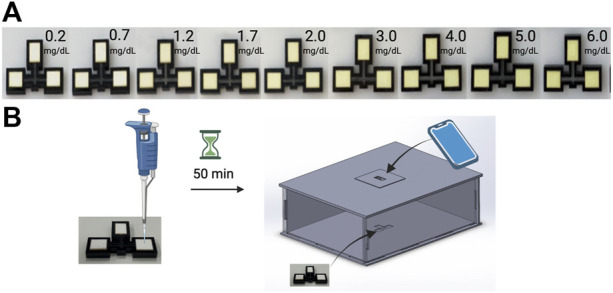
Quantification of bilirubin using kromaHealth Kit. **(A)** The color gradient observed in the panel with increasing concentrations of Bilirubin as it reacts with the stabilized reagents on the pads. **(B)** The process followed to quantify these colorimetric changes start with the pipetting of serum on the pad. After 50 min of waiting for the reaction that produces the color change, the device is placed inside the box and the camera is aligned with the pinhole for the image to be captured and processed for quantification.

**FIGURE 3:**
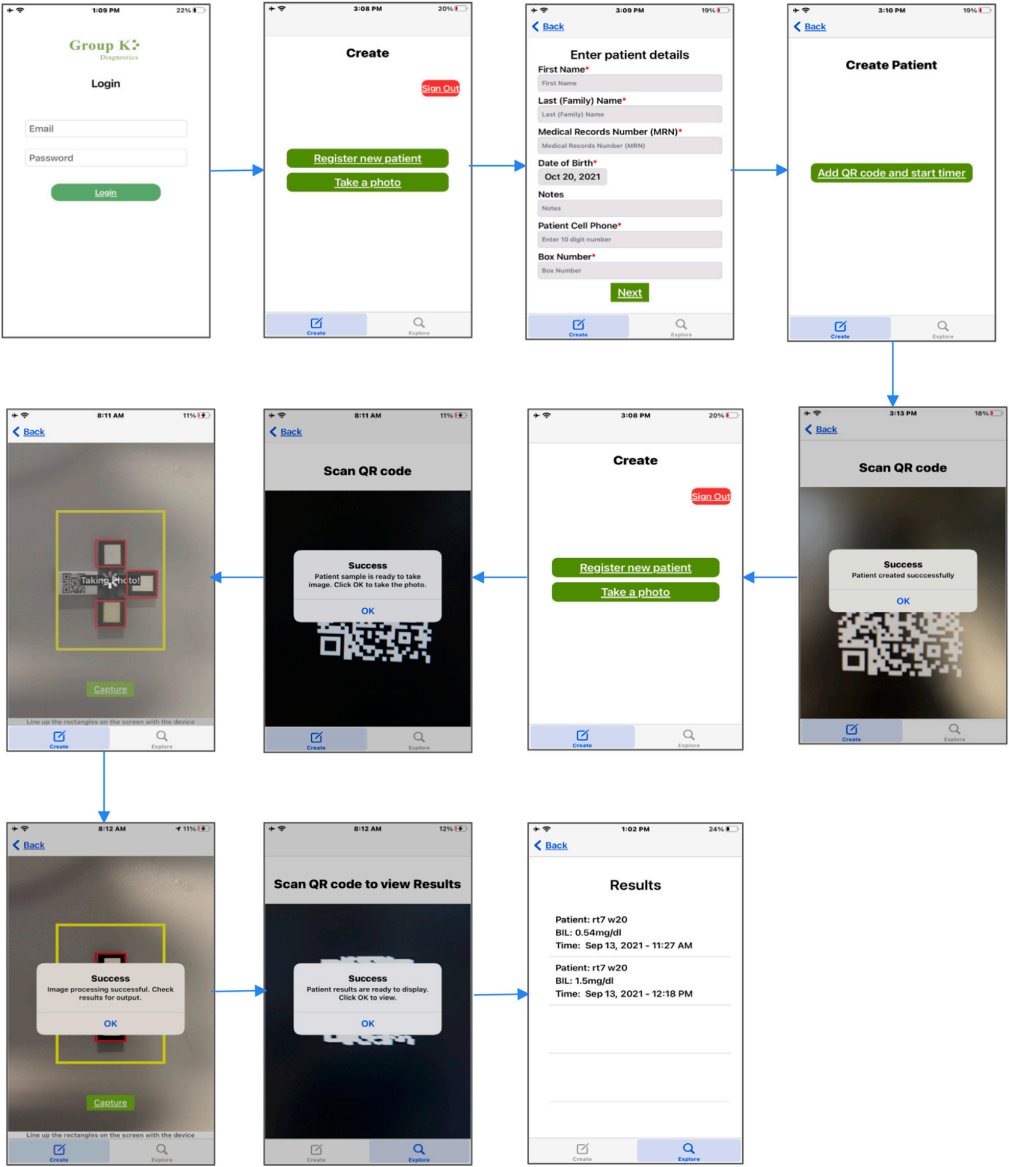
GKD iOS aplication workflow. The GKD application allows patients to be registered based on unique QR codes that are attached to an extension of the device. Once the patient is registered and the reaction process is complete, the application allows you to take a photo after scanning the QR code. The image is captured, and the selected ROI runs through the algorithm to predict a quantitative value for bilirubin. This is saved in the database and can be viewed again by scanning the patient QR code.

To develop a model which could predict the concentration of bilirubin in a patient’s serum sample from the color change of our assay devices, images of samples of known concentrations were collected. Figure 2 demonstrates the product workflow and therefore also how images of samples of known concentrations were generated to develop a model. Figure 2A shows a concentration gradient with increasing concentration of bilirubin which is clearly visible. Figure 2B shows how the serum is pipetted and how the colorimetric quantification is carried out using the phone and box. The overall process is demonstrated in Supplementary Video S1. Figure 3 shows how the phone app is used to take images of the device.

As discussed in Section 2.3 the ROI subimages need to be extracted from the images of the whole assay device. An algorithm was developed using OpenCV which could reliably crop the image down to just the desired ROI, a flow chart of this methodology is shown in Supplementary Figure S2. Once selected the ROI sub-images were extracted, duplicated into the RGB, XYZ, LAB, and HSV color spaces, and statistical features were collected on the color channel values which make up those color spaces, Supplementary Figure S3 shows this workflow. The statistical features collected include the mean, median, and mode of each of the color channels for each ROI. Supplementary Figure S4 shows the results of this process for a small set of 15 training images to demonstrate the responses of the various features collected. The dataset used for model training included more than 100 individual images of devices with samples having known bilirubin concentrations.

Linear and polynomial regression forms of the model were evaluated based on their R-squared values and tested against a validation set of images which were not used for training. It can be seen from Supplementary Figure S4 that even a linear model could yield quite high R-squared values, since the color channel statistics detailed in the figure show such a strong correlation to known bilirubin concentrations. These trends also demonstrate the consistency and accuracy of the ROI selection algorithm, since it must be selecting the areas of color change for this trend to be observed. Due to the proprietary nature of our model the exact details of its form and which channels were selected will not be disclosed. The model chosen was of a low order (less than 4), used color space features from only a single-color space, and did not use all of the statistical features calculated for that color space. The resulting model had a good R-squared value of 0.983. This model was used for all of the testing done in later sections of this paper.

### 3.2 Precision

A summary of the mean values and intraday, inter-day and inter-site variability for five measurements of bilirubin are shown in Table 1. The analysis yielded estimates for three variance components namely V_error_ (within-day) (Eq. 1), V_day_ (day to day within site) (Eq. 2) and V_site_ (between sites) (Eq. 3), wherein *MS* stands for Mean Square, and *n* stands for the number of days or replicates (rep).

**TABLE 1:** Two-way nested ANOVA-Variance components for the multi-site precision study.

Sample	Mean	V error (within day)	V day (between-day)	V site (between-site)
P1 (0.4 mg/dl)	0.39	0.012	0.001	0.001
P2 (0.2 mg/dl)	0.34	0.022	0.003	0.00
P3 (0.98 mg/dl)	1.20	0.100	0.004	0.001
P4 (1.5 mg/dl)	1.60	0.110	0.016	0.001
P5 (2.0 mg/dl)	2.30	0.250	0.021	0.003

The equations used to analyze the results are shown below, wherein *V* stands for variance, *MS* for mean square and *n* as number of replicates or days.

$$V_{error}=MS_{error}$$ (1)

$$V_{day}=\frac{\left(MS_{day}-MS_{error}\right)}{n_{rep}}$$ (2)

$$V_{site}=\frac{\left(MS_{site}-MS_{day}\right)}{n_{rep}n_{day}}$$ (3)

All the samples P1 through P5 do not show much variance from site to site over the days of the study. Standard deviation (SD) and % Covariance (%CV) is calculated for repeatability, within-laboratory precision, and reproducibility in Table 2. These values give the device’s precision profile. The repeatability (within-day precision) SD corresponds to V_error,_ within-laboratory precision corresponds to V_error_ and V_day,_ and the reproducibility (between-site precision) corresponds to all three- V_error,_ V_day,_ and V_site._ Of all the samples, P2 is showing the highest variation. The value of P2 is 0.2 mg/dl and it lies close to our lowest limit of detection. This could be the reason why the detection of low samples could vary in its prediction of results which increases the variance. The entire data set of the concentration of bilirubin for the multi-site precision study are given in Supplementary Table S1.

**TABLE 2:** Precision estimates: Overall and site by site for the multi-site precision study.

Sample	Mean (mg/dl)	Repeatability		Within-laboratory precision		Reproducibility	
		SD	%CV	SD	%CV	SD	%CV
P1	0.39	0.110	28.1	0.120	29.6	0.120	30.6
P2	0.34	0.150	43.5	0.160	46.7	0.160	46.7
P3	1.20	0.320	26.9	0.320	27.4	0.320	27.5
P4	1.60	0.330	20.8	0.360	22.2	0.360	22.3
P5	2.30	0.500	21.5	0.520	22.4	0.520	22.5
P1	0.52	0.14	25.90	0.14	27.5	Site 1	
P2	0.35	0.16	44.90	0.17	48.1		
P3	1.10	0.23	21.60	0.25	23.2		
P4	1.50	0.09	5.50	0.02	1.4		
P5	2.30	0.45	19.40	0.47	20.3		
P1	0.32	0.08	26.20	0.09	28	Site 2	
P2	0.33	0.13	39.70	0.14	43.1		
P3	1.20	0.35	29.00	0.36	29.6		
P4	1.70	0.19	11.20	0.02	0.97		
P5	2.40	0.56	23.10	0.57	23.5		
P1	0.33	0.08	23.00	0.08	24.5	Site 3	
P2	0.34	0.16	45.10	0.17	48.3		
P3	1.30	0.35	28.30	0.36	28.5		
P4	1.60	0.06	3.90	0.01	0.7		
P5	2.20	0.48	21.50	0.51	23.1		

Similarly, a summary of the statistics is listed in Tables 3, 4 for the single-site study. The repeatability is given V_error_, the within-laboratory precision is given by V_error,_ V_run_ (Eq. 4), and V_day_ (Eq. 5). Three samples out of 10 samples show less precision (high %CV) within the laboratory testing. The other samples show very minor variation. This lack of precision for some samples could have occurred due to various external factors like operator error, lighting condition, etc. The entire data set of the concentration of bilirubin for the single-site precision study are given in Supplementary Table S2.

**TABLE 3:** Statistical Summary for single site precision.

Sample description	Mean value (mg/dl)	V_error_	V_run_	V_day_
^Sample A (0.2 mg/dl)^	^0.30^	^0.01^	^0.00^	^0.00^
^Sample B (0.4 mg/dl)^	^0.49^	^0.02^	^0.00^	^0.01^
^Sample C (0.97 mg/dl)^	^0.79^	^0.11^	^0.04^	^0.02^
^Sample D (1.50 mg/dl)^	^1.60^	^0.41^	^0.41^	^0.04^
^Sample E (2.0 mg/dl)^	^2.10^	^0.20^	^0.14^	^0.84^
^Sample F (3.3 mg/dl)^	^3.90^	^1.40^	^1.20^	^0.17^
^Sample G (4.0 mg/dl)^	^4.60^	^1.60^	^1.30^	^0.94^
^Sample H (5.6 mg/dl)^	^6.40^	^1.90^	^1.00^	^0.34^
^Sample I (6.50 mg/dl)^	^6.40^	^1.50^	^0.88^	^0.16^
^Sample J (7.4 mg/dl)^	^7.30^	^0.47^	^0.45^	^0.06^

**TABLE 4:** SD and %CV for bilirubin precision in the single-site study.

^Sample description^	^Mean value^	^Repeatability^		^Within-laboratory precision^	
		^SD^	^%CV^	^SD^	^%CV^
^Sample A^	^0.30^	^0.11^	^36.6^	^0.09^	^30.0^
^Sample B^	^0.49^	^0.13^	^26.5^	^0.14^	^28.6^
^Sample C^	^0.79^	^0.33^	^41.8^	^0.41^	^51.9^
^Sample D^	^1.60^	^0.64^	^39.8^	^0.93^	^57.8^
^Sample E^	^2.10^	^0.45^	^21.7^	^1.10^	^52.7^
^Sample F^	^3.90^	^1.20^	^30.0^	^1.70^	^41.9^
^Sample G^	^4.60^	^1.30^	^27.3^	^2.00^	^42.2^
^Sample H^	^6.40^	^1.40^	^21.5^	^1.80^	^28.2^
^Sample I^	^6.40^	^1.20^	^19.0^	^1.60^	^24.8^
^Sample J^	^7.30^	^0.69^	^9.4^	^0.99^	^13.5^

The precision profiles for both studies are plotted for better visualization of the data summary and can be seen in Supplementary Figure S5. Despite some of the high %CV, all predicted values were acceptable within CLIA (Clinical Laboratory Improvement Amendments) set limits of ±0.4  mg/dl for total bilirubin.

$$V_{run}=\frac{\left(MS_{run}-MS_{error}\right)}{n_{rep}}$$ (4)

$$V_{day}=\frac{\left(MS_{day}-MS_{run}\right)}{n_{run}n_{rep}}$$ (5)

### 3.3 Interference

The results demonstrating the interfering substances for the assay of bilirubin at low and high concentrations are presented in Supplementary Tables S3A,B. d_obs-_ Point Estimate of interference effect is the difference of the mean of the test samples and mean value of the control samples. The criteria for declaring that a substance is an interferant is determined by the level of percentage interference. If the interference is below 10%, it is not considered as a potential interfering substance. Any presence of hemoglobin or hemolyzed samples interfered with the bilirubin testing in both high and low levels. Hemoglobin concentrations over 20 mg/dl have been known to contribute to the serum appearing hemolyzed (Dasgupta and Wahed, 2014). The hemoglobin interference is significantly high since it changes the color of the serum. Triglyceride levels up to 1,000 mg/dl did not interfere with low level samples, whereas triglyceride levels above 300 mg/dl interfered with high levels of bilirubin. Hence, we can conclude that if the test sample has a triglyceride value above 300 mg/dl it is marked to interfere with the bilirubin detection. Ascorbic acid levels up to 1.75 mg/dl did not interfere with high- or low-level bilirubin samples. Acetaminophen levels up to 5.20 mg/dl did not interfere with either level of bilirubin. Ibuprofen level below 21.90 mg/dl did not interfere up to 0.2 mg/dl of bilirubin samples whereas ibuprofen level above 13 mg/dl interfered with the detection of bilirubin at 2 mg/dl. Therefore, presence of ibuprofen below 13 mg/dl will not interfere with the detection of bilirubin.

### 3.4 Limits of Detection

The bilirubin levels to establish the LoB for Reagent Lots 1 and 2 are shown in Supplementary Tables S4A,B, respectively. The LoB was calculated using non-parametric analysis. Rank position was calculated for each lot. Rank position is calculated using Eq. 6 below, wherein *B* stands for the number of blank measurements per reagent lot and $Pct_{B}$ stands for the corresponding percentile (0.95 calculated using Type I error risk of α=0.05 )

$$Rank\;Position=0.5+B*Pct_{B}$$ (6)

Rank positions are integer values and interpolated as 21 and 22 for Lot 1 and 23 and 24 for Lot 2. Arranging the blank results in ascending order, LoB is the value at the calculated rank position. Both the lots for LoB were calculated individually (Table 5). The anomaly in Lot 1 values for Blank 1 was not considered for the calculations and attributed to experimental error such as pipetting or improper exposure.

**TABLE 5:** Rank positions and LoB from blank samples test results.

Rank position_Lot1	Value (lot 1)	Rank position_Lot2	Value (lot 2)
21	0.350	22	0.350
22	0.39	23	0.350
LoB	0.37		0.35

The highest LoB (0.37 mg/dl) was used to calculate the LoD (Table 6). For LoD, Lot 1 is calculated using 75 samples and Lot 2 uses 50 samples. The concentrations used to determine LoD are given in Supplementary Tables S5A,B. The LoD is calculated using Eq. 7 below, wherein $c_{p}$ is the multiplier that gives the 95th percentile of the normal distribution and $SD_{L}$ is the pooled SD per reagent lot.

$$LoD=c_{p}SD_{L}$$ (7)

**TABLE 6:** SDs and LoD calculations from low level sample test results.

Sample	Reagent Lot 1		Reagent lot 2	
	*n*	SD	*n*	SD
Low 1	15	0.05	10	0.03
Low 2	15	0.08	10	0.06
Low 3	15	0.07	10	0.09
Low 4	15	0.08	10	0.07
Low 5	15	0.06	10	0.08
SDl		0.07		0.069
cp		1.651		1.654
LoD		0.48		0.48

**FIGURE 4:**
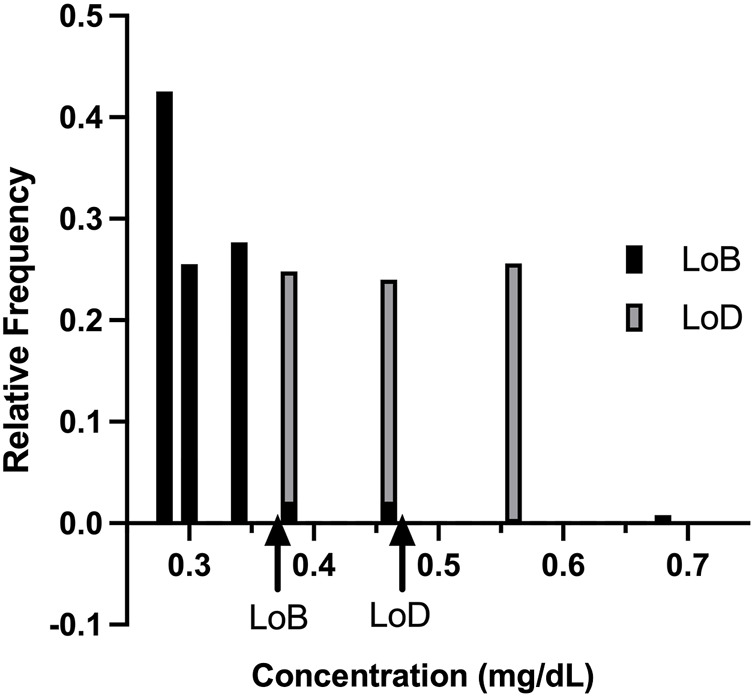
Relative frequency distribution. The relative frequency distribution for the limit of blank studies and limit of detection studies, as expected, shows minimal overlap in the concentration detected by the KromaHealth Kit.

Figure 4 shows the relative frequency distribution of LoB and LoD. The LoD for bilirubin was 0.48 mg/dl. This result is consistent with the guidelines in CLSI document EP17 based on the proportions of false positives (*α*) less than 5% and false negatives (*β*) less than 5% with 150 low level samples and 48 blank samples, and LoB of 0.37 mg/dl.

### 3.5 Linearity

**FIGURE 5:**
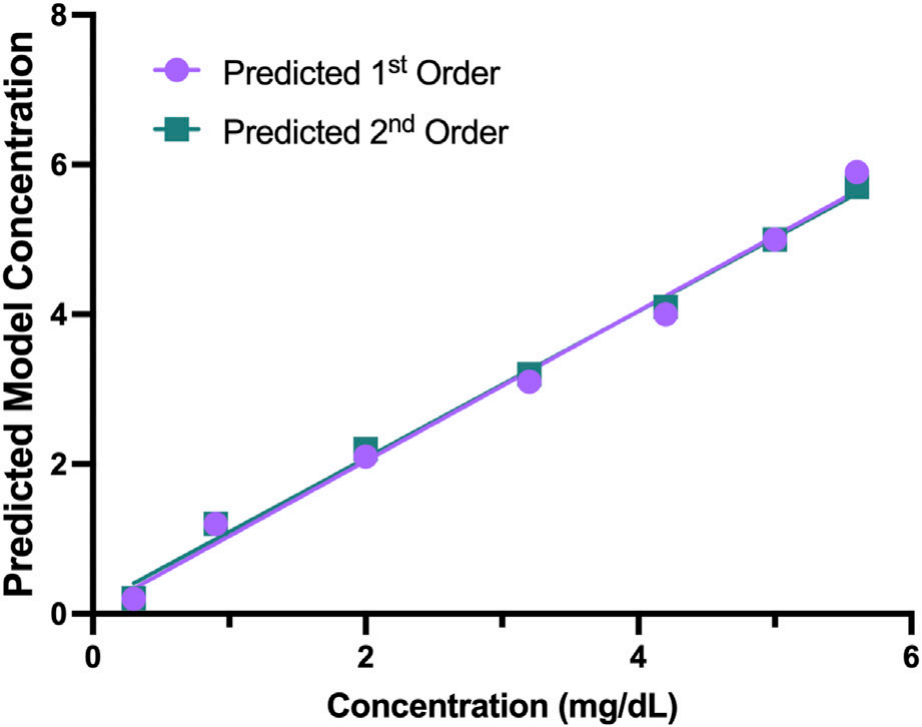
Predicted 1^st^ and 2^nd^ Order Models. The predicted first and second order models had no non-linear components, and the plot shows the predicted concentration for the two models.

The concentrations for each sample and the differences between the replicates with the KromaHealth are shown in Supplementary Tables S6A,B, respectively. Linearity using regression analysis is shown in Table 7. To prove linearity, the non-linearity of the system was evaluated. The non-linear coefficients were analyzed which are b2 in second order polynomial regression and b2 and b3 in third order polynomial regression. The second order did not have any non-linear components and the system was linear. In the third order fitting, one coefficient exceeds the criterion of 2.228 for 10 degrees of freedom. The second order model has a much lower standard error than the third order fit which proves that first order and second order prove better fitting than the third order. The predicted results for the first and second order polynomial regression are presented in Table 8. The percentage difference was less than the laboratory criterion of 20% which proves that the system is linear. The linearity plot can be seen in Figure 5.

**TABLE 7:** Regression analysis.

Order	Co ef. Symbol	Coefficient value	Coefficient SE	*t*-test	Degrees freedom	Std. Error regression
First	b_0_	0.69	0.19	3.64	12	0.225
	b_1_	0.93	0.04	23.35		
Second	b_0_	−1.00	0.35	−2.87	11	0.225
	b_1_	1.14	0.2	5.71		
	**b**_**2**_	−**0.03**	**0.02**	−**1.25**		
Third	b_0_	0.06	0.14	0.44	10	0.449
	b_1_	−0.07	0.14	−0.51		
	**b**_**2**_	**0.33**	**0.04**	**8.23**		
	**b**_**3**_	−**0.03**	**0.03**	−**0.97**		

b2 and b3 are coefficients in the second order and third order models, respectively. The columns show the average value of the coefficients calculated using the data, their standard error and t-test for regression to test for correlation.

**TABLE 8:** Predicted results with 1st order and 2nd order predictions.

Result mean	Predicted 1st order	Predicted 2nd order	Difference	%Difference
0.31	0.24	0.21	−0.03	−15.00
0.94	1.20	1.20	0.00	0.01
2.00	2.10	2.20	0.08	3.60
3.20	3.00	3.20	0.10	3.30
4.20	4.00	4.10	0.08	1.90
5.10	4.90	4.90	0.00	0.02
5.60	5.90	5.70	−0.13	−2.20

### 3.6 Comparison With Predicate Device

**FIGURE 6:**
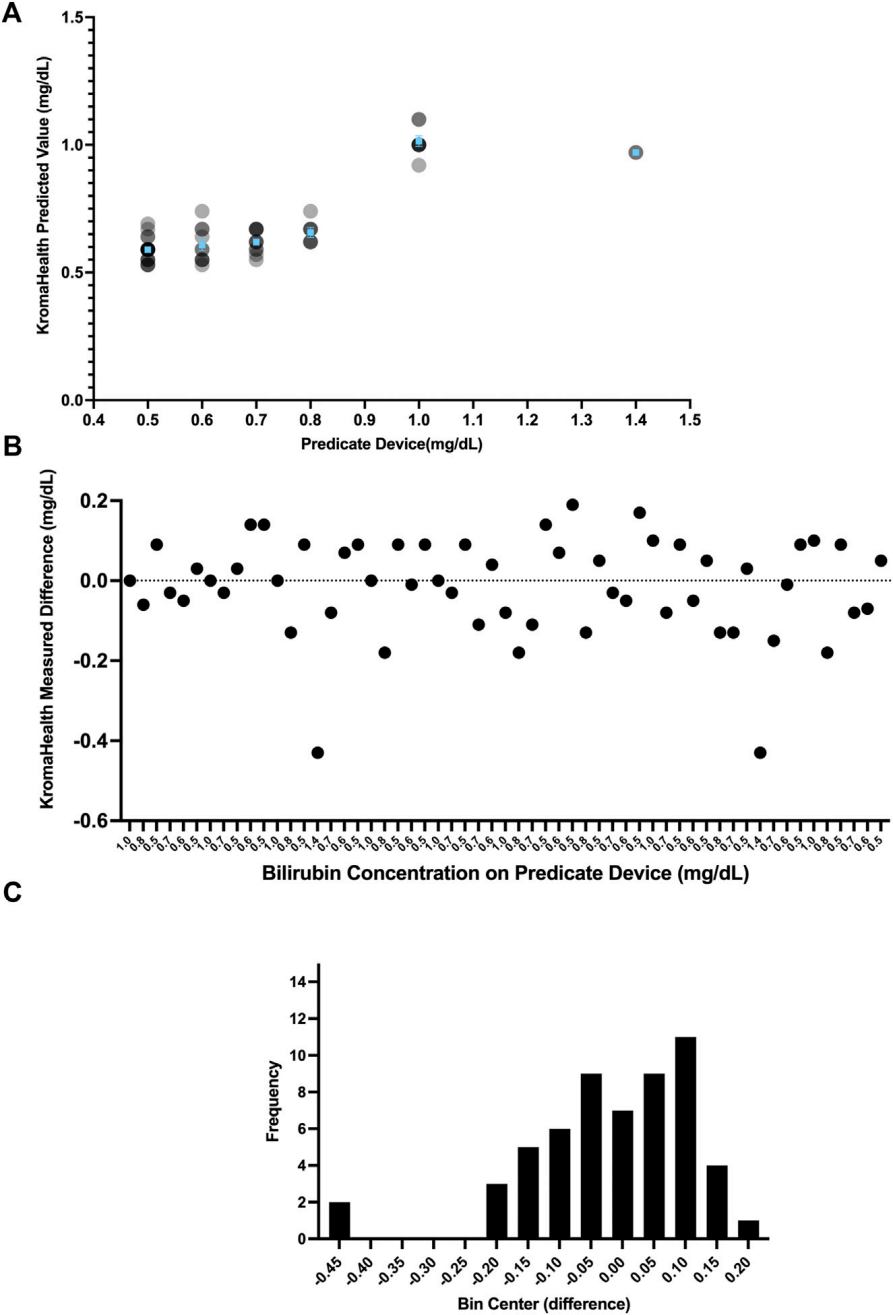
Performance compared to predicate device. Various distributions of the comparison studies can be seen. **(A)** The individual read-outs are plotted for each concentration tested in the predicate device and KromaHealth kit. Identical concentration readouts overlap when plotted and appear as darker points with increasing overlaps. The mean and standard error of the device is plotted in blue for each concentration tested. **(B)** Each point here shows the difference between a single read-out and its corresponding known concentration from the predicate device. **(C)** The skewed frequency distribution of the differences is seen here, and this is used to calculate the bias estimate.

The data for the predicate device and KromaHealth kit is shown in Supplementary Table S7. This data showing the predicted KromaHealthKit results for the predicate value is plotted in Figure 6A. The difference plot for each sample is shown in Figure 6B and the distribution of the difference is shown in Figure 6C. The difference value is calculated by taking the difference between predicate concentration value KromaHealth kit predicted value for each sample. The average percentage error for the samples that were measured was 13.46% and error ranged from 0% to 30%. For all bilirubin samples less than or equal to 1 mg/dl, the percentage error was less than 13%.

Since the difference has a skewed vertical distribution, bias estimation was computed from the median of difference values as 0 mg/dl for the measured range of 0.5–1.4 mg/dl. The bias (of measurement) is defined as the estimation of a systematic measurement error between the test result and the predicate reference value. The 95% CI for this bias is calculated using the Wilcoxon Signed Rank Test as −0.05 to 0.05 mg/dl. The predefined bias criterion for equivalence was set to be ± 0.1 mg/dl. Therefore, the criterion for equivalence was met for the range of concentrations measured.

### 3.7 Shelf-Life Study

**FIGURE 7:**
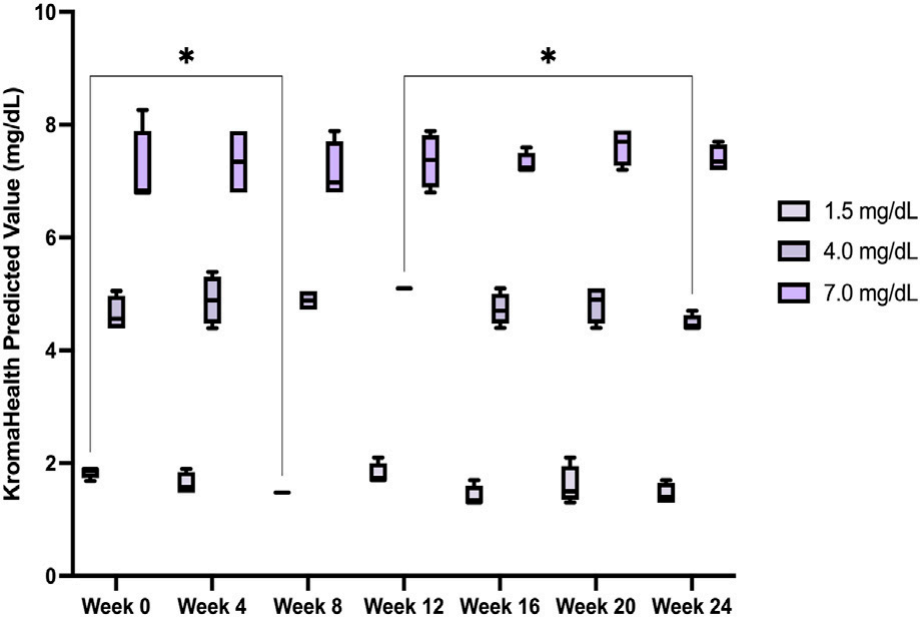
Device stability over a 6-month period. The device performance was measured over a period of 6 months and found to be satisfactory, without significant deviation from the original known concentrations of the samples studied.

Results from the 6-month shelf-life study showed no significant (*p* < 0.05) changes within the samples over the 24 weeks, except between Week 0 and Week 8 for 1.5 mg/dl and Week 12 and Week 24 for 4 mg/dl (Figure 7). Over the 6-month period, average concentrations and standard error for each sample was 1.5 ±0.05 mg/dl, 4.8 ± 0.06 mg/dl, and 7.3 ± 0.1 mg/dl. However, slight changes in the predicted values could be due to the variation in serum sample aliquots used for the study. The results show the device with the spotted reagents are stable at least up to 6 month at room temperature. Data from the study is shown in Supplementary Figure S8.

## 4 Conclusion

In conclusion, we developed an efficient and inexpensive paper-based device for colorimetric quantification of bilirubin using a phone camera and backend image processing algorithms. We were able to demonstrate reliable precision levels, a real-time 6-month shelf-life, and equivalence in terms of performance when compared to the Roche cobalt c311, an exorbitantly priced predicate. Therefore, due to its low-costs, ease of use, and with a short 50 min turnover time, the device will be useful in POC and clinics, especially in limited-resource settings. Additionally, the image processing and machine learning technology platform to quantify colorimetric biochemical processes can be applied to measure various biomarker levels for diagnostic and prognostic purposes. Future studies will focus on carrying forward this technology to develop more tests useful for liver function, infectious diseases and cancer.

## Data Availability

The original contributions presented in the study are included in the article/Supplementary Material, further inquiries can be directed to the corresponding authors.
