# Supplementary material for: A Low-Cost Paper-Based Device for the Colorimetric Quantification of Bilirubin in Serum Using Smartphone Technology
Source: Front Chem. 2022 Jul 7;10:869086. doi: 10.3389/fchem.2022.869086 (PMC9301313; doi:10.3389/fchem.2022.869086)
Supplement: Supplementary file 2 [file DataSheet1.docx]

Supplementary Material

Low-cost paper-based device for the colorimetric quantification of bilirubin in serum using smartphone technology

Brittany AuYoung^1^, Akshay Ravichandran^2^, Divykumar Patel^2^, Nisarg Dave^1^, Achal Shah^1^, Brianna Wronko^3^, Franklin Bettencourt^1^, Reshma Rajan^1+*^, Nidhi Menon^1+*^

^1^Division of Product Development, Group K Diagnostics, Philadelphia, PA, USA

^2^Manufacturing Department, Group K Diagnostics, Philadelphia, PA, USA

^3^Board of Directors, Group K Diagnostics, Philadelphia, PA, USA

**
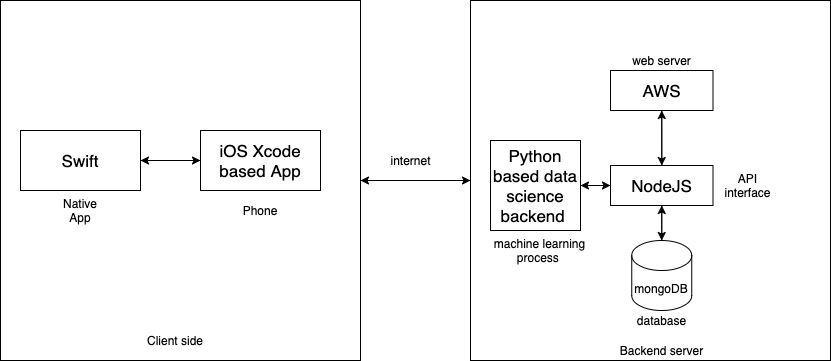
**

**Supplementary Figure 1. Stack Overview**


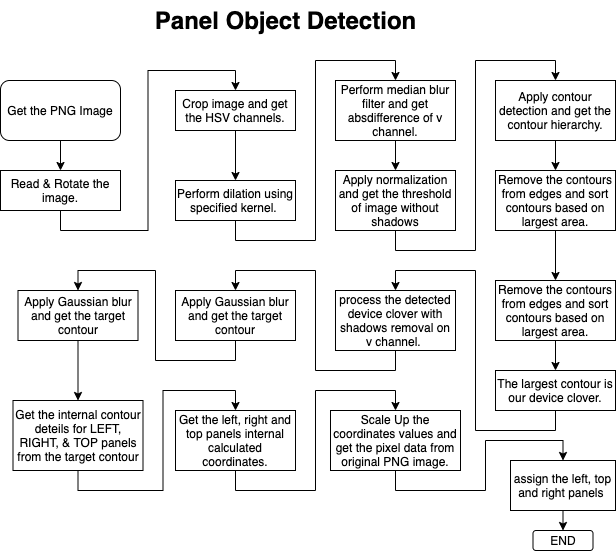


**Supplementary Figure 2. Image Processing Workflow.**

**
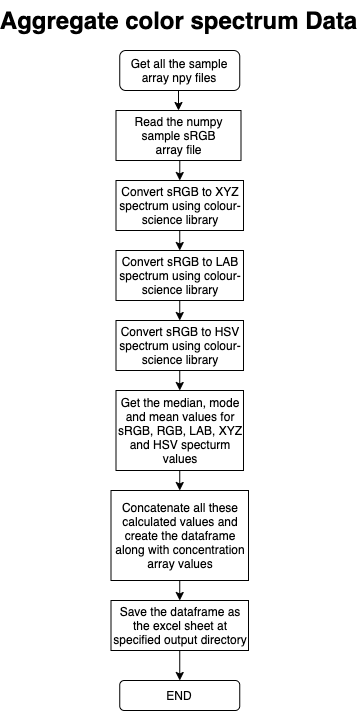
**

**Supplementary Figure 3. Workflow for calculating the color spectrum arrays.**


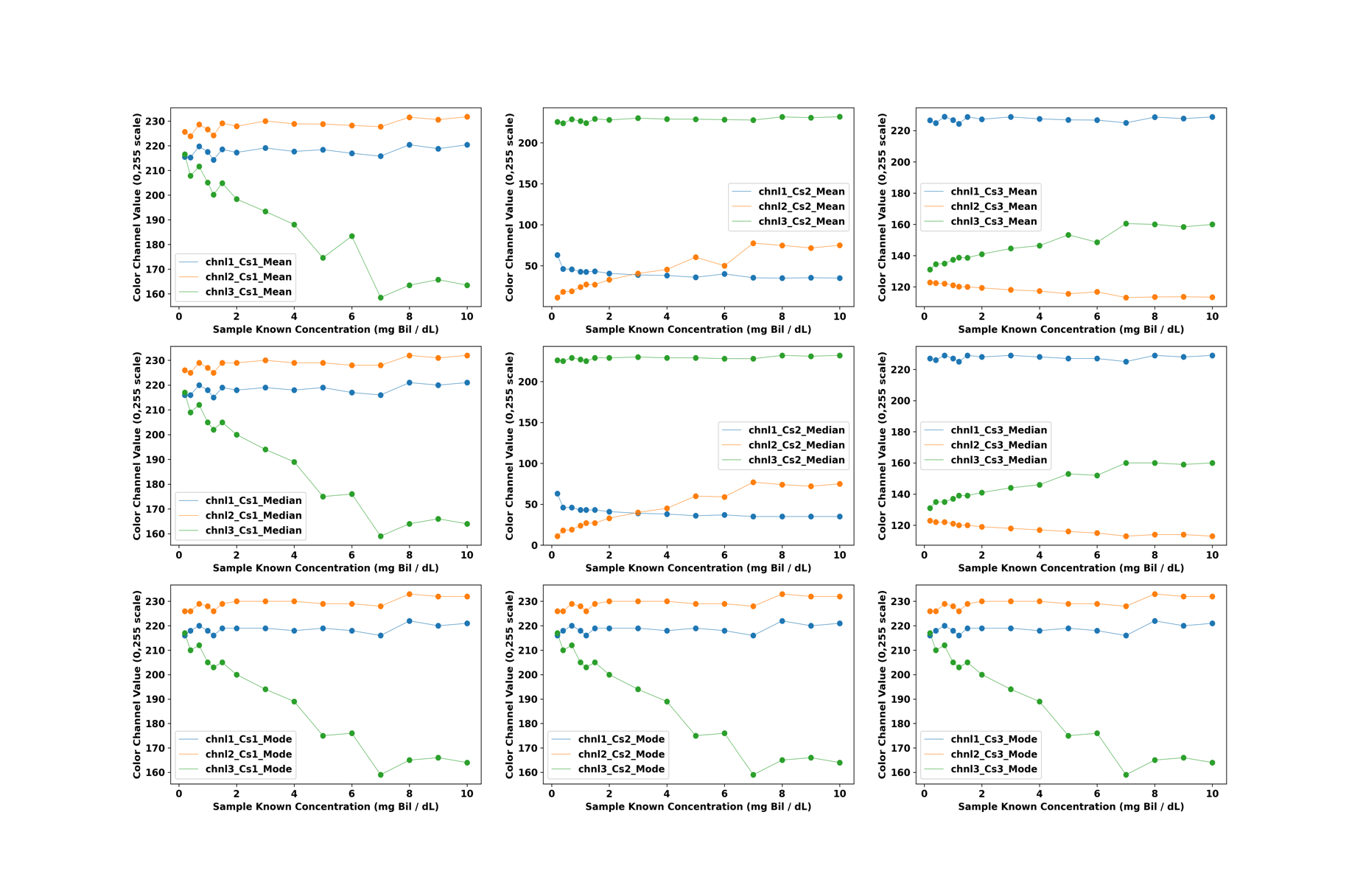


**Supplementary Figure 4.**  Results from plotting different statistical features for a small subset of training images. The plots are split out into a column for each color space and a row for each statistic of interest. The curves are each labeled using a prefix for the channel index of the color space, followed by the color space, and then a suffix with the statistic the curve is plotting. chnl1_Cs1_Mean would refer to the first channel of color space 1 and the mean values for all of the pixels in each image in the subset. The color spaces included here are the HSV, LAB, and RGB spaces. Each point on a curve represents a single image of known concentration of Bilirubin (mg / dL).

**Supplementary Table 1. Data set for the multi-site precision study.**

| **Day** | **Sample** | **Site1** | **Site 2** | **Site 3** | **Sample** | **Site1** | **Site 2** | **Site 3** | **Sample** | **Site1** | **Site 2** | **Site 3** |
| --- | --- | --- | --- | --- | --- | --- | --- | --- | --- | --- | --- | --- |
| 1 | P1 | 0.46 | 0.29 | 0.39 | P3 | 1.10 | 1.10 | 1.10 | P5 | 1.90 | 2.60 | 1.30 |
| 1 | P1 | 0.46 | 0.33 | 0.33 | P3 | 0.67 | 1.10 | 1.10 | P5 | 1.90 | 2.60 | 2.40 |
| 1 | P1 | 0.56 | 0.29 | 0.33 | P3 | 0.94 | 1.10 | 1.30 | P5 | 2.90 | 2.40 | 2.10 |
| 1 | P1 | 0.56 | 0.33 | 0.33 | P3 | 0.94 | 1.10 | 0.94 | P5 | 1.90 | 2.60 | 2.10 |
| 1 | P1 | 0.56 | 0.33 | 0.39 | P3 | 0.94 | 0.94 | 0.94 | P5 | 2.90 | 2.40 | 1.90 |
| 2 | P1 | 0.56 | 0.29 | 0.33 | P3 | 1.10 | 1.70 | 1.10 | P5 | 1.90 | 3.20 | 1.50 |
| 2 | P1 | 0.56 | 0.29 | 0.29 | P3 | 0.94 | 1.50 | 0.94 | P5 | 1.90 | 2.60 | 2.40 |
| 2 | P1 | 0.56 | 0.29 | 0.29 | P3 | 0.94 | 1.70 | 0.94 | P5 | 1.90 | 2.90 | 1.90 |
| 2 | P1 | 0.46 | 0.29 | 0.28 | P3 | 0.94 | 1.50 | 1.10 | P5 | 1.90 | 3.20 | 2.10 |
| 2 | P1 | 0.56 | 0.33 | 0.29 | P3 | 0.94 | 1.70 | 1.30 | P5 | 2.10 | 2.60 | 2.40 |
| 3 | P1 | 0.46 | 0.33 | 0.33 | P3 | 1.10 | 1.30 | 1.30 | P5 | 2.90 | 2.60 | 2.10 |
| 3 | P1 | 0.56 | 0.33 | 0.33 | P3 | 1.10 | 0.94 | 1.30 | P5 | 2.60 | 2.90 | 2.60 |
| 3 | P1 | 0.56 | 0.39 | 0.29 | P3 | 0.94 | 1.50 | 1.30 | P5 | 2.60 | 2.10 | 2.40 |
| 3 | P1 | 0.56 | 0.33 | 0.33 | P3 | 0.94 | 1.10 | 1.30 | P5 | 2.60 | 1.90 | 2.10 |
| 3 | P1 | 0.46 | 0.28 | 0.39 | P3 | 0.80 | 0.94 | 1.30 | P5 | 2.60 | 2.10 | 1.90 |
| 4 | P1 | 0.56 | 0.39 | 0.33 | P3 | 1.30 | 1.70 | 1.10 | P5 | 2.10 | 2.90 | 2.10 |
| 4 | P1 | 0.67 | 0.33 | 0.39 | P3 | 1.30 | 1.10 | 1.30 | P5 | 2.60 | 2.40 | 1.90 |
| 4 | P1 | 0.56 | 0.33 | 0.33 | P3 | 1.30 | 1.10 | 1.10 | P5 | 2.10 | 2.40 | 2.40 |
| 4 | P1 | 0.56 | 0.33 | 0.33 | P3 | 0.94 | 0.94 | 1.50 | P5 | 2.10 | 2.40 | 2.60 |
| 4 | P1 | 0.56 | 0.33 | 0.29 | P3 | 1.30 | 0.80 | 1.30 | P5 | 1.90 | 1.90 | 2.10 |
| 5 | P1 | 0.46 | 0.33 | 0.33 | P3 | 1.30 | 1.10 | 1.50 | P5 | 2.10 | 1.90 | 2.40 |
| 5 | P1 | 0.46 | 0.29 | 0.33 | P3 | 1.30 | 0.94 | 1.30 | P5 | 2.40 | 1.90 | 2.40 |
| 5 | P1 | 0.46 | 0.29 | 0.33 | P3 | 1.70 | 1.10 | 1.50 | P5 | 2.60 | 1.90 | 2.40 |
| 5 | P1 | 0.46 | 0.33 | 0.33 | P3 | 1.10 | 1.10 | 1.90 | P5 | 2.40 | 1.90 | 2.10 |
| 5 | P1 | 0.46 | 0.33 | 0.33 | P3 | 1.30 | 0.94 | 1.70 | P5 | 2.40 | 1.90 | 3.80 |
| 1 | P2 | 0.28 | 0.33 | 0.39 | P4 | 0.94 | 1.70 | 1.10 |  |  |  |  |
| 1 | P2 | 0.28 | 0.29 | 0.29 | P4 | 0.94 | 1.70 | 1.70 |  |  |  |  |
| 1 | P2 | 0.29 | 0.29 | 0.33 | P4 | 1.50 | 1.70 | 1.30 |  |  |  |  |
| 1 | P2 | 0.28 | 0.29 | 0.29 | P4 | 1.50 | 1.90 | 1.70 |  |  |  |  |
| 1 | P2 | 0.28 | 0.29 | 0.28 | P4 | 0.94 |  | 1.70 |  |  |  |  |
| 2 | P2 | 0.33 | 0.39 | 0.29 | P4 | 1.50 | 1.70 | 1.70 |  |  |  |  |
| 2 | P2 | 0.33 | 0.33 | 0.29 | P4 | 1.50 | 1.30 | 1.50 |  |  |  |  |
| 2 | P2 | 0.33 | 0.31 | 0.29 | P4 | 1.50 | 1.50 | 1.90 |  |  |  |  |
| 2 | P2 | 0.33 | 0.33 | 0.33 | P4 | 1.50 | 1.50 | 1.90 |  |  |  |  |
| 2 | P2 | 0.33 | 0.33 | 0.29 | P4 | 1.50 | 1.50 | 1.50 |  |  |  |  |
| 3 | P2 | 0.33 | 0.33 | 0.56 | P4 | 1.50 | 2.40 | 1.30 |  |  |  |  |
| 3 | P2 | 0.33 | 0.33 | 0.33 | P4 | 1.50 | 2.40 | 1.30 |  |  |  |  |
| 3 | P2 | 0.33 | 0.33 | 0.33 | P4 | 1.50 | 1.70 | 1.50 |  |  |  |  |
| 3 | P2 | 0.33 | 0.29 | 0.33 | P4 | 2.10 | 1.50 | 1.30 |  |  |  |  |
| 3 | P2 | 0.39 | 0.33 | 0.33 | P4 | 1.50 | 1.10 | 1.50 |  |  |  |  |
| 4 | P2 | 0.33 | 0.29 | 0.39 | P4 | 2.10 | 1.50 | 1.30 |  |  |  |  |
| 4 | P2 | 0.46 | 0.29 | 0.33 | P4 | 1.70 | 2.10 | 1.70 |  |  |  |  |
| 4 | P2 | 0.46 | 0.29 | 0.39 | P4 | 1.50 | 1.50 | 1.70 |  |  |  |  |
| 4 | P2 | 0.39 | 0.33 | 0.39 | P4 | 1.50 | 1.30 | 1.90 |  |  |  |  |
| 4 | P2 | 0.33 | 0.29 | 0.39 | P4 | 1.50 | 1.30 | 1.70 |  |  |  |  |
| 5 | P2 | 0.46 | 0.39 | 0.33 | P4 | 1.70 | 2.10 | 1.70 |  |  |  |  |
| 5 | P2 | 0.39 | 0.33 | 0.39 | P4 | 1.50 | 2.40 | 1.90 |  |  |  |  |
| 5 | P2 | 0.33 | 0.39 | 0.39 | P4 | 1.50 | 2.10 | 1.30 |  |  |  |  |
| 5 | P2 | 0.39 | 0.39 | 0.33 | P4 | 1.90 | 1.90 | 1.70 |  |  |  |  |
| 5 | P2 | 0.33 | 0.33 | 0.33 | P4 | 1.90 | 2.10 | 1.90 |  |  |  |  |

**Supplementary Table 2. Single-site precision study**

|  | Run1 (mg/dL) | |  | Run 2 (mg/dL) | |
| --- | --- | --- | --- | --- | --- |
| Sample concentration (mg/dL) | Rep1 | Rep 2 | Sample concentration (mg/dL) | Rep1 | Rep 2 |
| 0.2 | 0.28 | 0.28 | 0.2 | 0.39 | 0.33 |
| 0.2 | 0.28 | 0.29 | 0.4 | 0.39 | 0.33 |
| 0.2 | 0.28 | 0.29 | 0.4 | 0.67 | 0.56 |
| 0.4 | 0.46 | 0.46 | 0.97 | 1.5 | 0.94 |
| 0.4 | 0.46 | 0.56 | 0.97 | 0.8 | 0.67 |
| 0.97 | 0.67 | 0.56 | 1.5 | 1.3 | 1.1 |
| 0.97 | 0.56 | 0.67 | 1.5 | 2.1 | 1.5 |
| 1.5 | 2.6 | 2.4 | 2.0 | 1.9 | 1.9 |
| 1.5 | 0.94 | 0.94 | 2.0 | 1.5 | 1.7 |
| 2.0 | 2.6 | 2.9 | 2.0 | 2.1 | 1.9 |
| 3.3 | 2.9 | 2.9 | 3.3 | 5.7 | 5.4 |
| 3.3 | 4.1 | 3.8 | 3.3 | 3.5 | 3.2 |
| 4 | 6.4 | 5.7 | 4 | 4.1 | 3.5 |
| 4 | 5.1 | 5.4 | 4 | 3.5 | 3.5 |
| 5.6 | 6.4 | 6.8 | 5.6 | 7.5 | 6.1 |
| 5.6 | 7.9 | 7.2 | 5.6 | 4.7 | 4.7 |
| 6.5 | 5.1 | 6.1 | 6.5 | 5.7 | 5.7 |
| 6.5 | 8.6 | 8.3 | 6.5 | 5.7 | 6.1 |
| 7.4 | 7.2 | 6.8 | 7.4 | 7.2 | 7.2 |
| 7.4 | 8.3 | 8.6 | 7.4 | 7.2 | 6.4 |

**
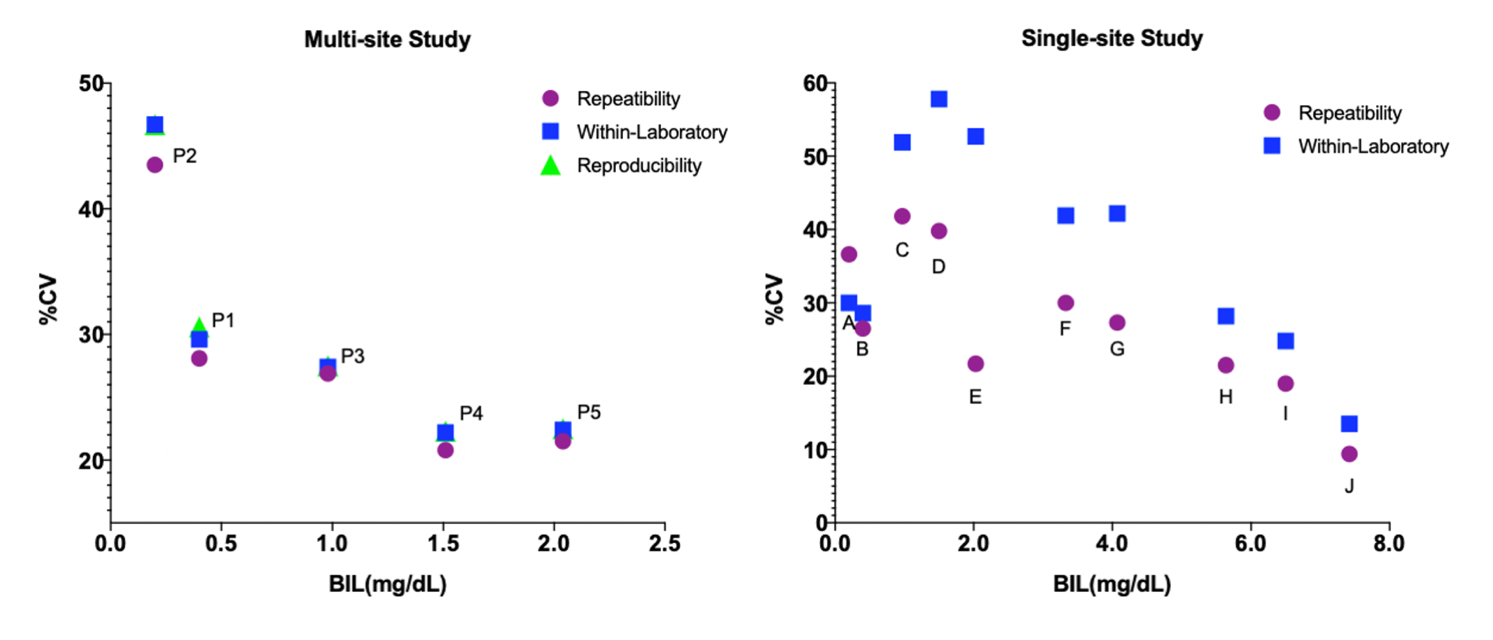
**

**Supplementary Figure 5. Comparison of Precision Estimates.** The precision estimates for multi-site and single-site studies are summarized by graphing the %CV as a function of the concentration of Bilirubin.

**Supplementary Table 3A: Interfering substances for low level bilirubin test**

| Low Sample (0.4 mg/dL) | Measured results, mg/dL | | | | | Mean, mg/dL | SD | Interference difference, d_obs_ | Interference percentage % |
| --- | --- | --- | --- | --- | --- | --- | --- | --- | --- |
|  | Rep 1 | Rep 2 | Rep 3 | Rep 4 | Rep 5 |  |  |  |  |
| Control | 0.39 | 0.46 | 0.46 | 0.39 | 0.33 | 0.41 | 0.06 | 3.33 | 821.18 |
| Hemoglobin (1000) | 3.50 | 3.80 | 3.50 | 3.80 | 4.10 | 3.74 | 0.25 |  |  |
|  |  |  |  |  |  |  |  |  |  |
| Low Sample (0.4 mg/dL) | Measured results, mg/dL | | | | | Mean, mg/dL | SD | Interference difference, d_obs_ | Interference percentage % |
|  | Rep 1 | Rep 2 | Rep 3 | Rep 4 | Rep 5 |  |  |  |  |
| Control | 0.39 | 0.38 | 0.39 | 0.39 | 0.39 | 0.39 | 0.00 | 0.02 | 4.64 |
| Triglyceride (1000) | 0.33 | 0.39 | 0.46 | 0.39 | 0.46 | 0.41 | 0.06 |  |  |
|  |  |  |  |  |  |  |  |  |  |
| Low Sample (0.4 mg/dL) | Measured results, mg/dL | | | | | Mean, mg/dL | SD | Interference difference, d_obs_ | Interference percentage % |
|  | Rep 1 | Rep 2 | Rep 3 | Rep 4 | Rep 5 |  |  |  |  |
| Control | 0.39 | 0.46 | 0.46 | 0.39 | 0.33 | 0.41 | 0.06 | 0.02 | 4.64 |
| Ascorbic Acid (1.75) | 0.39 | 0.46 | 0.39 | 0.56 | 0.33 | 0.43 | 0.09 |  |  |
|  |  |  |  |  |  |  |  |  |  |
| Low Sample (0.4 mg/dL) | Measured results, mg/dL | | | | | Mean, mg/dL | SD | Interference difference, d_obs_ | Interference percentage % |
|  | Rep 1 | Rep 2 | Rep 3 | Rep 4 | Rep 5 |  |  |  |  |
| Control | 0.39 | 0.46 | 0.46 | 0.39 | 0.33 | 0.41 | 0.06 | -0.03 | -7.47 |
| Acetaminophen (5.20) | 0.33 | 0.39 | 0.39 | 0.39 | 0.39 | 0.38 | 0.03 |  |  |
|  |  |  |  |  |  |  |  |  |  |
| Low Sample (0.4 mg/dL) | Measured results, mg/dL | | | | | Mean, mg/dL | SD | Interference difference, d_obs_ | Interference percentage % |
|  | Rep 1 | Rep 2 | Rep 3 | Rep 4 | Rep 5 |  |  |  |  |
| Control | 0.39 | 0.46 | 0.46 | 0.39 | 0.33 | 0.41 | 0.06 | -0.04 | -9.35 |
| Acetylsalicylic acid (3.00) | 0.39 | 0.46 | 0.33 | 0.33 | 0.33 | 0.37 | 0.06 |  |  |
|  |  |  |  |  |  |  |  |  |  |
| Low Sample (0.4 mg/dL) | Measured results, mg/dL | | | | | Mean, mg/dL | SD | Interference difference, d_obs_ | Interference percentage % |
|  | Rep 1 | Rep 2 | Rep 3 | Rep 4 | Rep 5 |  |  |  |  |
| Control | 0.39 | 0.46 | 0.46 | 0.39 | 0.33 | 0.41 | 0.06 | -0.01 | -1.90 |
| Ibuprofen (21.90) | 0.39 | 0.33 | 0.33 | 0.56 | 0.39 | 0.40 | 0.09 |  |  |

**Supplementary Table 3B: Interfering Substances for high level bilirubin test**

| High Sample (2.0 mg/dL) | Measured results, mg/dL | | | | | Mean, mg/dL | SD | Interference difference, d_obs_ | Interference percentage % |
| --- | --- | --- | --- | --- | --- | --- | --- | --- | --- |
|  | Rep 1 | Rep 2 | Rep 3 | Rep 4 | Rep 5 |  |  |  |  |
| Control | 2.40 | 2.40 | 2.10 | 2.10 | 2.40 | 2.28 | 0.16 | 1.76 | 77.19 |
| Hemoglobin (1000) | 4.10 | 3.80 | 4.10 | 4.10 | 4.10 | 4.04 | 0.13 |  |  |
|  |  |  |  |  |  |  |  |  |  |
| High Sample (2.0 mg/dL) | Measured results, mg/dL | | | | | Mean, mg/dL | SD | Interference difference, d_obs_ | Interference percentage % |
|  | Rep 1 | Rep 2 | Rep 3 | Rep 4 | Rep 5 |  |  |  |  |
| Control | 2.10 | 2.40 | 2.30 | 2.10 | 2.40 | 2.26 | 0.15 | -0.06 | -2.65 |
| Triglyceride (300) | 2.60 | 2.10 | 2.10 | 2.10 | 2.10 | 2.20 | 0.22 |  |  |
|  |  |  |  |  |  |  |  |  |  |
| High Sample (2.0 mg/dL) | Measured results, mg/dL | | | | | Mean, mg/dL | SD | Interference difference, d_obs_ | Interference percentage % |
|  | Rep 1 | Rep 2 | Rep 3 | Rep 4 | Rep 5 |  |  |  |  |
| Control | 2.40 | 2.40 | 2.10 | 2.10 | 2.40 | 2.28 | 0.16 | 0.20 | 8.77 |
| Ascorbic Acid (1.75) | 2.10 | 2.40 | 2.90 | 2.90 | 2.10 | 2.48 | 0.40 |  |  |
|  |  |  |  |  |  |  |  |  |  |
| High Sample (2.0 mg/dL) | Measured results, mg/dL | | | | | Mean, mg/dL | SD | Interference difference, d_obs_ | Interference percentage % |
|  | Rep 1 | Rep 2 | Rep 3 | Rep 4 | Rep 5 |  |  |  |  |
| Control | 2.40 | 2.40 | 2.10 | 2.10 | 2.40 | 2.28 | 0.16 | 0.20 | 8.77 |
| Acetaminophen (5.20) | 3.20 | 2.10 | 2.10 | 2.40 | 2.60 | 2.48 | 0.45 |  |  |
|  |  |  |  |  |  |  |  |  |  |
| High Sample (2.0 mg/dL) | Measured results, mg/dL | | | | | Mean, mg/dL | SD | Interference difference, d_obs_ | Interference percentage % |
|  | Rep 1 | Rep 2 | Rep 3 | Rep 4 | Rep 5 |  |  |  |  |
| Control | 2.40 | 2.40 | 2.10 | 2.10 | 2.40 | 2.28 | 0.16 | -0.06 | -2.63 |
| Acetylsalicylic acid (3.00) | 2.10 | 2.40 | 2.10 | 1.90 | 2.60 | 2.22 | 0.28 |  |  |
|  |  |  |  |  |  |  |  |  |  |
| High Sample (2.0 mg/dL) | Measured results, mg/dL | | | | | Mean, mg/dL | SD | Interference difference, d_obs_ | Interference percentage % |
|  | Rep 1 | Rep 2 | Rep 3 | Rep 4 | Rep 5 |  |  |  |  |
| Control | 2.40 | 2.40 | 2.10 | 2.40 | 2.30 | 2.32 | 0.13 | 0.12 | 5.17 |
| Ibuprofen (13.00) | 2.40 | 2.60 | 2.40 | 2.40 | 2.40 | 2.44 | 0.09 |  |  |

**Supplementary Table 4. Predicted values for LoB Measurement**

**(A)Lot 1**

| **Day** | **Replicate** | **Blank 1** | **Blank 2** | **Blank 3** | **Blank 4** |
| --- | --- | --- | --- | --- | --- |
| 1 | 1 | 0.35 | 0.3 | 0.3 | 0.28 |
|  | 2 | 0.35 | 0.35 | 0.28 | 0.28 |
| 2 | 1 | 1.7 | 0.39 | 0.28 | 0.35 |
|  | 2 | 0.3 | 0.46 | 0.28 | 0.35 |
| 3 | 1 | 0.3 | 0.28 | 0.33 | 0.28 |
|  | 2 | 0.28 | 0.28 | 0.28 | 0.28 |

**(A)Lot 2**

| **Day** | **Replicate** | **Blank 1** | **Blank 2** | **Blank 3** | **Blank 4** |
| --- | --- | --- | --- | --- | --- |
| 1 | 1 | 0.35 | 0.3 | 0.28 | 0.28 |
|  | 2 | 0.35 | 0.35 | 0.28 | 0.3 |
| 2 | 1 | 0.35 | 0.28 | 0.29 | 0.35 |
|  | 2 | 0.3 | 0.29 | 0.28 | 0.35 |
| 3 | 1 | 0.29 | 0.28 | 0.28 | 0.28 |
|  | 2 | 0.3 | 0.3 | 0.28 | 0.33 |

**Supplementary Table 5. Predicted values for LoD Measurement**

**(A)Lot 1**

| **Day** | **Replicate** | **Low 1** | **Low 2** | **Low 3** | **Low 4** | **Low 5** |
| --- | --- | --- | --- | --- | --- | --- |
| 1 | 1 | 0.29 | 0.33 | 0.39 | 0.33 | 0.56 |
|  | 2 | 0.33 | 0.33 | 0.39 | 0.46 | 0.46 |
|  | 3 | 0.29 | 0.39 | 0.39 | 0.39 | 0.46 |
| 2 | 1 | 0.33 | 0.39 | 0.39 | 0.46 | 0.39 |
|  | 2 | 0.33 | 0.33 | 0.39 | 0.39 | 0.46 |
|  | 3 | 0.33 | 0.33 | 0.39 | 0.39 | 0.39 |
| 3 | 1 | 0.46 | 0.39 | 0.39 | 0.56 | 0.56 |
|  | 2 | 0.46 | 0.46 | 0.46 | 0.46 | 0.56 |
|  | 3 | 0.39 | 0.39 | 0.46 | 0.56 | 0.56 |
| 4 | 1 | 0.29 | 0.39 | 0.56 | 0.56 | 0.56 |
|  | 2 | 0.33 | 0.46 | 0.56 | 0.56 | 0.56 |
|  | 3 | 0.33 | 0.56 | 0.46 | 0.56 | 0.56 |
| 5 | 1 | 0.33 | 0.33 | 0.46 | 0.46 | 0.46 |
|  | 2 | 0.33 | 0.33 | 0.56 | 0.56 | 0.56 |
|  | 3 | 0.29 | 0.56 | 0.56 | 0.46 | 0.46 |
|  |  |  |  |  |  |  |

**(B) Lot 2**

|  | **Replicate** | **Low 1** | **Low 2** | **Low 3** | **Low 4** | **Low 5** |
| --- | --- | --- | --- | --- | --- | --- |
| **Day** | 1 | 0.33 | 0.39 | 0.39 | 0.46 | 0.46 |
|  | 2 | 0.33 | 0.29 | 0.33 | 0.39 | 0.39 |
| 2 | 1 | 0.29 | 0.39 | 0.39 | 0.39 | 0.46 |
|  | 2 | 0.33 | 0.33 | 0.39 | 0.46 | 0.46 |
| 3 | 1 | 0.29 | 0.39 | 0.56 | 0.39 | 0.56 |
|  | 2 | 0.39 | 0.39 | 0.56 | 0.56 | 0.56 |
| 4 | 1 | 0.33 | 0.46 | 0.46 | 0.56 | 0.67 |
|  | 2 | 0.33 | 0.46 | 0.56 | 0.56 | 0.56 |
| 5 | 1 | 0.33 | 0.39 | 0.46 | 0.46 | 0.46 |
|  | 2 | 0.29 | 0.46 | 0.56 | 0.56 | 0.56 |

**Supplementary Table 6A: Bilirubin Assay results at 7 Concentrations**

| Sample Number | Rep 1 (mg/dL) | Rep 2 (mg/dL) | Mean (mg/dL) |
| --- | --- | --- | --- |
| 1 (0.2 mg/dL) | 0.29 | 0.33 | 0.31 |
| 2 (1.02 mg/dL) | 0.94 | 0.94 | 0.94 |
| 3 (1.79 mg/dL) | 1.70 | 2.40 | 2.00 |
| 4 (2.61 mg/dL) | 3.20 | 3.20 | 3.20 |
| 5 (3.43 mg/dL) | 4.10 | 4.40 | 4.20 |
| 6 (4.19 mg/dL) | 5.10 | 5.10 | 5.10 |
| 7 (5.01 mg/dL) | 5.70 | 5.40 | 5.60 |

**Supplementary Table 6B: Difference in bilirubin concentrations between each replicate set**

| Sample Number | Mean | Difference (d) | (d2/2) |
| --- | --- | --- | --- |
| 1 | 0.31 | 0.04 | 0.001 |
| 2 | 0.94 | 0 | 0.000 |
| 3 | 2.00 | 0.7 | 0.245 |
| 4 | 3.20 | 0 | 0.000 |
| 5 | 4.20 | 0.3 | 0.045 |
| 6 | 5.10 | 0 | 0.000 |
| 7 | 5.60 | -0.3 | 0.045 |

**Supplementary Table 7. Method of Comparison**

| Predicate Device (mg/dL) | KromaHealth Kit Predicted Result (mg/dL) |
| --- | --- |
| 1 | 1.00 |
| 0.8 | 0.74 |
| 0.5 | 0.59 |
| 0.7 | 0.67 |
| 0.6 | 0.55 |
| 0.5 | 0.53 |
| 1 | 1.00 |
| 0.7 | 0.67 |
| 0.5 | 0.53 |
| 0.6 | 0.74 |
| 0.5 | 0.64 |
| 1 | 1.00 |
| 0.8 | 0.67 |
| 0.5 | 0.59 |
| 1.4 | 0.97 |
| 0.7 | 0.62 |
| 0.6 | 0.67 |
| 0.5 | 0.59 |
| 1 | 1.00 |
| 0.8 | 0.62 |
| 0.5 | 0.59 |
| 0.6 | 0.59 |
| 0.5 | 0.59 |
| 1 | 1.00 |
| 0.7 | 0.67 |
| 0.5 | 0.59 |
| 0.7 | 0.59 |
| 0.6 | 0.64 |
| 1 | 0.92 |
| 0.8 | 0.62 |
| 0.7 | 0.59 |
| 0.5 | 0.64 |
| 0.6 | 0.67 |
| 0.5 | 0.69 |
| 0.8 | 0.67 |
| 0.5 | 0.55 |
| 0.7 | 0.67 |
| 0.6 | 0.55 |
| 0.5 | 0.67 |
| 1 | 1.10 |
| 0.7 | 0.62 |
| 0.5 | 0.59 |
| 0.6 | 0.55 |
| 0.5 | 0.55 |
| 0.8 | 0.67 |
| 0.7 | 0.57 |
| 0.5 | 0.53 |
| 1.4 | 0.97 |
| 0.7 | 0.55 |
| 0.6 | 0.59 |
| 0.5 | 0.59 |
| 1 | 1.10 |
| 0.8 | 0.62 |
| 0.5 | 0.59 |
| 0.7 | 0.62 |
| 0.6 | 0.53 |
| 0.5 | 0.55 |

**Supplementary Table 8: KromaHealth Predicted Bilirubin concentrations for the 6-month shelf-life study**

|  | **1. 5 mg/dL** | | | | **4.0 mg/dL** | | | | **7.0 mg/dL** | | | |
| --- | --- | --- | --- | --- | --- | --- | --- | --- | --- | --- | --- | --- |
| **Week 0** | 1.9 | 1.9 | 1.7 | 1.9 | 5.1 | 4.7 | 4.4 | 4.4 | 6.8 | 6.8 | 8.3 | 6.8 |
| **Week 4** | 1.7 | 1.9 | 1.5 | 1.5 | 4.7 | 5.4 | 5.1 | 4.4 | 6.8 | 7.9 | 6.8 | 7.9 |
| **Week 8** | 1.5 | 1.5 | 1.5 | 1.5 | 5.1 | 4.7 | 4.7 | 5.1 | 7.9 | 6.8 | 7.2 | 6.8 |
| **Week 12** | 2.1 | 1.7 | 1.7 | 1.7 | 5.1 | 5.1 | 5.1 | 5.1 | 6.8 | 7.9 | 7.6 | 7.2 |
| **Week 16** | 1.3 | 1.3 | 1.7 | 1.3 | 5.1 | 4.7 | 4.7 | 4.4 | 7.6 | 7.2 | 7.2 | 7.2 |
| **Week 20** | 1.3 | 2.1 | 1.5 | 1.5 | 5.1 | 5.1 | 4.4 | 4.7 | 7.2 | 7.5 | 7.9 | 7.9 |
| **Week 24** | 1.3 | 1.5 | 1.7 | 1.3 | 4.4 | 4.4 | 4.7 | 4.4 | 7.2 | 7.7 | 7.5 | 7.2 |

**
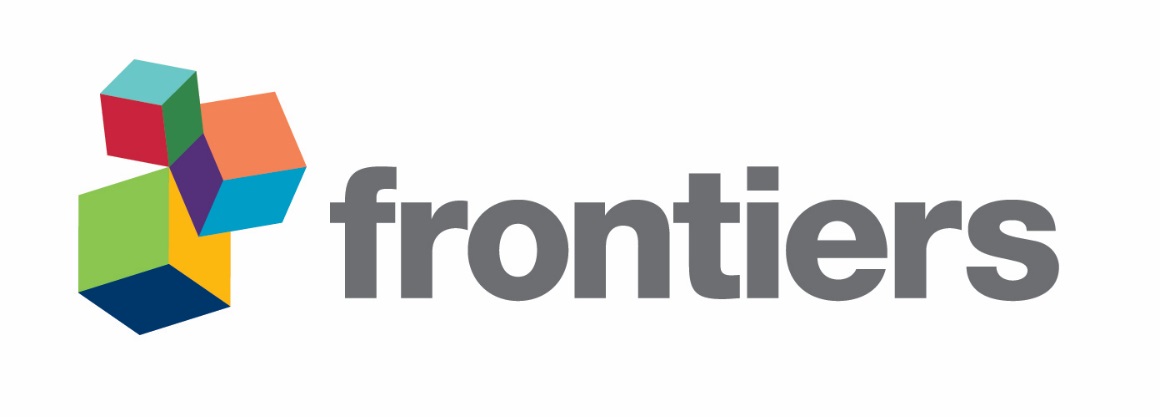
**
